# Applicability of Small and Low-Cost Magnetic Sensors to Geophysical Exploration

**DOI:** 10.3390/s24217047

**Published:** 2024-10-31

**Authors:** Filippo Accomando, Giovanni Florio

**Affiliations:** Department of Earth, Environmental and Resources Sciences, University of Naples “Federico II”, 80126 Naples, Italy; gflorio@unina.it

**Keywords:** geophysical sensors, scalar magnetometers, vector magnetometers, UAV magnetic survey

## Abstract

In the past few decades, there has been a notable technological advancement in geophysical sensors. In the case of magnetometry, several sensors were used, having the common feature of being miniaturized and lightweight, thus idoneous to be carried by UAVs in drone-borne magnetometric surveys. A common feature is that their sensitivity ranges from 0.1 to about 200 nT, thus not comparable to that of optically pumped, standard fluxgate or even proton magnetometers. However, their low cost, volume and weight remain very interesting features of these sensors. In fact, such sensors have the common feature of being very inexpensive, so new ways of making surveys using many of these sensors could be devised, in addition to the possibility, even with limited resources, of creating gradiometers by combining two or more of them. In this paper, we explore the range of applicability of small tri-axial magnetometers commonly used for attitude determination in several devices. We compare the results of surveys performed with standard professional geophysical instruments with those obtained using these sensors and find that in the presence of strongly magnetized sources, they succeeded in identifying the main anomalies.

## 1. Introduction

Magnetic investigations aim to measure the Earth’s magnetic field, which varies in the presence of magnetized rocks containing ferrous minerals or in the presence of structures and objects of anthropic origin. These rocks or objects are sources of an induced secondary magnetic field, which can be sufficient to cause a measurable increase or decrease in the local magnetic field. The magnetic method therefore represents a non-invasive, rapid and inexpensive tool that plays an important role in different geophysical applications. For example, magnetic surveys have long been used for mapping archaeological remains, buried igneous or volcanic structures, mineral deposits, large geological structures and geological limits between different formations (in some cases indicative of faults) (e.g., [1,2,3]). They might also be useful for locating buried objects such as military unexploded ordnance, metallic drums, pipes and cables, and in other engineering research (e.g., [4,5]).

In the past few decades, the introduction of UAVs (unmanned aerial vehicles) and the development of miniaturized and light magnetic sensors have opened new possibilities for the acquisition of magnetic data (e.g., [6,7,8,9]). In fact, drone magnetometry allows the collection of magnetic datasets characterized by uniform coverage and a good resolution. In addition, drone magnetometry allows for reducing costs, times and risks compared to other acquisition techniques and allows for performing surveys on places that are difficult to access, for example in the presence of dense vegetation, cultivated areas, marshes, glaciers, lakes, areas of shoreline or steep mountain slopes.

Leaving aside a complete description of the working principles of the magnetic sensors, for which we refer the readers to more specific textbooks and papers (e.g., [10]), we now describe the main characteristics of magnetometers classically used in geophysical exploration and of sensors commonly used in other contexts and sometimes proposed for some geophysical applications.

### 1.1. Magnetic Sensors

The characteristics of magnetic sensors can give rise to different types of magnetometers. In Table 1, the sensors are presented according to some general features (dimensions, the capability to perform continuous or intermittent measurements, etc.) and two fundamental characteristics, namely the sample rate and the sensitivity. The sample rate may be important for qualifying the magnetometer as able to be carried on board a fast vehicle while still maintaining a good spatial sampling of the magnetic field. Moreover, a high sampling rate might be essential to correctly sample high-frequency magnetic signals generated by power lines’ AC signals and by the drone motors (if used in drone-borne surveys), and thus be able to correctly remove this noise. Finally, the sensitivity of a magnetometer sensor is a statistical value indicating the uncertainty of repetitive readings of the same magnetic field intensity, and it is expressed as the RMS value per square root of a unit of bandwidth (Hz^1/2^; e.g., [11]). Sensitivity is related to the Signal-to-Noise Ratio (SNR), as it defines the smallest change in the magnetic field that can be detected. The sensitivity varies according to the sampling frequency so that each sensor can be characterized by its sensitivity envelope, defining the increase in the value of sensitivity with the increase in the sampling rate.

### 1.2. Scalar and Vector Magnetometers

The different magnetic field sensors can be classified according to the quantity actually measured so that they can be distinguished into scalar and vector magnetometers.

The scalar magnetometers measure the magnitude of the total field, regardless of its vector direction. The total field includes the amplitude of the main Earth field as well as that of the secondary fields generated, by induction or by the presence of remanent magnetization, by other magnetized sources present in the crust. Time-varying fields generated outside the Earth (e.g., ionosphere) are removed with the help of a second magnetometer monitoring the variation in the magnetic field at a specific site in or near the survey area. By subtracting the scalar intensity of the Earth’s magnetic field (represented by the International Geomagnetic Reference Field model, IGRF) from the scalar intensity of the total magnetic field, we obtain the total field anomaly (e.g., [12]). This simple scalar difference well approximates the intensity of the component of the anomalous magnetic field in the direction of the Earth’s field, if the anomalous field is small with respect to the Earth’s magnetic field. In fact, if the anomalous field has an intensity lower than 1000 nT and the Earth’s magnetic field intensity is 50,000 nT, the error of this approximation is less than 1% (e.g., [13]).

The most common scalar magnetometers used in geophysical applications are the proton precession (also in the version exploiting the Overhauser effect) and optically pumped sensors. The proton precession magnetometers are practically insensitive to any orientation of the sensor, so their use in the field is very easy. The proton precession sensors are nowadays used essentially in the Overhauser version, allowing sensitivity and sampling frequency suitable for local surveys needing the detection of weak signals from small objects and structures.

The optically pumped magnetometers can use different alkali vapors in their optical cell (e.g., Cesium, Potassium, Rubidium). This type of magnetometer is relatively independent of the sensor orientation, but little or no signal may be generated if the optical axis of the sensor is lined up within a certain angle with the Earth’s magnetic vector (“dead zones”). Thus, some attention to the sensor orientation is needed, both in the planning of the direction of the survey lines and in their use in the field. Optically pumped magnetometers offer much better performances than proton precession magnetometers in terms of sensitivity and sampling rate. In addition, they have recently been miniaturized, with a sensor weight of only a few tens of grams, making this sensor type perfectly suitable for drone-borne magnetic surveys (e.g., [14]).

Different from scalar sensors, vector magnetometers detect a specific component of the Earth’s magnetic field along the direction with which the sensor is aligned. Vector magnetometry offers additional information about the sensed field with respect to scalar magnetometers. For example, vector measurements accurately represent the crustal magnetic fields, regardless of the strength of the anomalous field. This is achieved at the cost of some operational difficulty, such as the need to keep the sensor in the same orientation throughout a measurement survey. When transported by vehicles or carried by hand, even small rotational vibrations may cause sensor misalignments, generating non-negligible changes in the vector components of the Earth’s magnetic field that may be difficult to separate from the signal (e.g., [15]). For example, in a geomagnetic field of 50,000 nT, a variation in the sensor orientation of only 0.001° can produce an error of about 1 nT in the measured vector components [16]. Inertial measurement units (IMUs) can be used to determine the changes in position in the three spatial dimensions during the measurements so that appropriate corrections can be applied. Other errors are associated with the non-orthogonality of the sensor and offset problems (e.g., [17]), whose correction requires further calibration procedures.

Vector magnetometers based on fluxgate sensors are widely used in geophysical exploration. Typically, these sensors are composed of two bars of a high-permeability ferromagnetic material wrapped by two windings, forming a primary, or excitation, coil and a secondary, or pick-up, coil that detects the changes in magnetic flux passing through the sensor. Fluxgate sensors are frequently arranged to form three-component (3C) magnetometers. Fluxgate sensors are also available arranged as a vertical gradiometer, where a single pair of sensors is oriented along the vertical direction. Miniaturized 3C versions of this magnetometer are suitable for drone magnetometry (e.g., [18]). Ref. [19] tested a commercial motion sensor, based on a triaxial fluxgate magnetometer, as a vector magnetometer towed behind a research vessel. Fluxgate sensors output is not stable with respect to temperature changes, exhibiting a drift of the order of 0.1 nT/C° (e.g., [11]).

Superconducting quantum interference device (SQUID) represent the most sensitive magnetic field sensors available today, although their adoption in exploration geophysics is still sporadic because of practical difficulties of their use. In fact, SQUID sensors are made with superconductors that have to be immersed in liquid helium in order to reach a working temperature as low as 4.2 K. Their sensitivity is the best among the geophysical sensors, about 2 × 10^−5^ nT for a bandwidth of 10 Hz [20]. These sensors are built as intrinsic gradiometers [21] having a baseline (the distance between the centers of the two sensing loops) of only 4 cm. Such a small baseline reduces the gradiometer resolution, but this negative effect is counterbalanced by the extremely high sensitivity of the SQUID sensors. Ref. [20] show the application of a measurement system based on SQUID sensors on an archaeological area, where it successfully detected subtle magnetic anomalies produced by a Neolithic ditch. In that case, magnetic data were acquired at a sampling rate of 1000 Hz with the sensors mounted on a cart pulled by a cross-country vehicle.

For the sake of completeness, we report here that very recently experimental results have been presented to demonstrate the possibility of obtaining vector magnetic data also using single-beam optically pumped magnetometers (e.g., [22]).

### 1.3. Small and Lightweight Magnetic Sensors

Other magnetometers have been introduced in the last decades, having interesting characteristics, such as a small dimension, a low weight, and a low price.

Ref. [23] documents the use of a small, lightweight and inexpensive magneto-inductive sensor in the magnetic exploration of an iron ore deposit in Iran. This type of sensor is formed by three coils perpendicular to each other, implementing a 3C magnetometer. The measurement principle is based on the computation of the time difference between the charge and discharge of an inductor, which directly relates to the magnetic strength applied to the sensor coil [24]. Ref. [23] arranged two of these magneto-inductive sensors to form a total field vertical gradiometer. Their results compared favorably to that of an Overhauser sensor, in the case of very intense magnetic anomalies (amplitudes >15,000 nT).

Another magnetometer having similar characteristics in terms of dimensions, weight and cost is based on Anisotropy Magnetoresistance (AMR) technology. It measures the magnetic intensity along three directions from the resistance change in the AMR resistors in response to variations in the external magnetic field. Magnetoresistance magnetometers are simply energized by applying a constant current and the output voltage is a direct measure of the magnetic field. This kind of 3C magnetometer is often used as a compass in other devices. For example, an AMR sensor is included in the control unit of the Geometrics Micro-Fabricated Atomic Magnetometer (MFAM) (an optically pumped magnetometer), serving as a compass in the inertial measurement units (IMU).

Probably the most widespread magnetometer type today is the one based on the Hall effect, as this type of magnetometer is widely used as a compass sensor in smartphones. These sensors measure the intensity of magnetic fields when a conductor is subjected to a magnetic field perpendicular to the current flowing through it. As a result, a voltage (known as the Hall voltage) develops across the conductor. The magnitude of this voltage is directly proportional to the strength of the magnetic field.

The sensitivity of both AMR and Hall-effect sensors is of the order of 150–200 nT at a sample frequency of 100 Hz.

In this paper, we want to explore the possibility of using AMR and Hall-effect sensors in a geophysical exploration context. Their intrinsic high noise floor will obviously limit their application to the study of intense anomalies, but their very low cost and widespread availability might make them an attractive alternative to standard high-quality sensors in some applications.

We are aware of only a few papers where low-cost sensors were tested in typical applied geophysics applications. Ref. [25] used a smartphone in a drone-borne survey to reveal the presence of metallic wellheads in Oklahoma. The survey successfully measured a 3000 nT monopolar anomaly generated by the vertical metallic case of the well when flying at 10 m above ground level (agl), but this strong anomaly was not more visible when the flight altitude increased to 20 m agl. Ref. [23] compared the performance of an inexpensive magneto-inductive sensor with an Overhauser magnetometer in a mining area in Iran. They found that the magneto-inductive sensor (in this case, a couple of these sensors were arranged to form a vertical gradiometer with a baseline of 70 cm) reproduced the anomaly measured by the Overhauser magnetometer (ranging from about 50,000 to 80,000 nT) within a ±400 nT error, with a mean around 0 nT. In that case, the inexpensive sensor demonstrated better stability with respect to strong horizontal gradients, in the central part of the surveyed area, where the Overhauser magnetometer could not obtain any measurement. Ref. [26] tested the same sensors in a vertical gradiometric configuration in a UAV survey of the same mining area in Iran. Ref. [27] tested the Hall-effect sensor contained in a mobile phone above steel pipes and above a landfill area. Their results were compared to an optically pumped magnetometer, and they found an overall acceptable performance of the Hall-effect sensor.

We will use an empirical approach for testing the actual possibility of using these low-cost sensors in real geophysical surveys by presenting results in a variety of applied geophysical applications. Ref. [28] tested Hall-effect magnetic sensors present inside two types of smartphones and found that the sampled data contained two kinds of artifacts: sudden “glitches” (spikes) having an amplitude as big as 1 μT, and longer-term DC shifts with amplitudes ranging from 2 to 5 μT. Our tests will verify the presence of these types of artifacts in the data acquired by AMR and Hall-effect sensors. However, we aim at a more complete characterization of their behavior in a geophysical survey, verifying also the stability of measurements in the presence of strong horizontal gradients and the characteristics of the noise affecting the real measurements.

**Table 1:** A non-exhaustive table of magnetic field sensors used, or potentially useful, in geophysics. For each sensor, its typical sensitivity for a specified bandwidth is reported.

Scalar Sensors	Sensitivity (nT/√Hz)	Sampling Rate	Dimension	Comments
Proton precession	0.15 @ 1 Hz	0.3–2 Hz	Bulky	intermittent readings; robust; independent of the orientation
Overhauser	0.015 @ 1 Hz	0.2–5 Hz	Bulky	high-sensitivity proton magnetometer with higher sampling rate (continuous)
Optically pumped (Cs, K, Rb)	10^−3^–10^−4^ @ 1 Hz	10–1000 Hz	From bulky to miniaturized	expensive; relatively fragile; needs some orientation (to account for dead zones)
**Vectorial sensors**	**Sensitivity (nT/** **√** **Hz)**	**Sampling rate**	**Dimension**	**Comments**
Fluxgate	0.1–0.01 @ 1 Hz	100 Hz–250 Hz (up to analog)	From bulky to miniaturized	needs orientation; subject to thermal drift
SQUID ^1^	2 × 10^−5^ @ 10 Hz	Up to 3 MHz	miniaturized	expensive; relatively fragile; needs orientation; requires cryogenic refrigeration, so that the complete system is bulky
Anisotropy Magneto-Resistance and Hall effect	150–200 @ 100 Hz	100 Hz (typical)	miniaturized	inexpensive; needs orientation; commonly used as compass sensors in smartphones or other devices
Magneto-inductive ^2^	2 @ 1 Hz 9 @ 40 Hz	Up to 40 Hz	miniaturized	inexpensive; needs orientation

^1^ Sensitivity and sampling rate data are relative to the implementation described in [20]. ^2^ Sensitivity and sampling rate data as described in [29].

## 2. Materials and Methods

In the next section, we compare the data acquired by two types of low-cost magnetic sensors with the MFAM high-sensitivity Cesium optically pumped sensor. The two low-cost sensors are an AMR magnetometer, used as a compass inside the MFAM control unit, and a Hall-effect magnetic sensor built inside an iPhone mobile phone for navigation and screen calibration purposes. Both are vector sensors, providing the measurement of 3 components of the total magnetic field along mutually perpendicular directions. On the contrary, the MFAM is an optically pumped magnetic sensor and provides a scalar measurement of the total field (as already mentioned in the Introduction).

In the following examples, all relative to strong magnetic fields generated by highly magnetized sources, the vector datasets can be conveniently compared to the scalar dataset if the intensity of the total magnetic field, *T*, is computed from the measured *X*, *Y* and *Z* components:

(1) $$T=\sqrt{\left(X^{2}+Y^{2}+Z^{2}\right)}$$

The intensity of the total magnetic field (as defined in Equation (1)) has the property of being invariant to rotations, solving the problem of the field variations measured at the three sensors generated even by small rotations of the 3C magnetometer.

As the examples of surveys presented below involve small areas, where the IGRF does not vary significantly and can be considered as a constant, the total field intensity data are presented without subtracting the main field.

## 3. Results

Below, we present the results obtained from three different surveys which involve both ground and UAV investigations.

### 3.1. Area 1

The first example application concerns a ground magnetic survey to test different sensors in a small uncultivated and abandoned area (“Area 1”) in Naples (Italy). In this area, some rebar metal rods outcrop here and there, so the diffused presence of metallic objects at depth is highly probable.

The survey covered an area of 40 × 28 m^2^, with profiles of varying lengths spaced 1 m apart, approximately oriented from north to south. Both the MFAM sensor and the Hall-effect sensor (contained in an iPhone 15) were carried at an approximately equal elevation of about 1 m above ground level (AGL).

We used the Phyphox free application to record the information from the Hall-effect magnetic sensor contained in the mobile phone. We recorded the data of each profile in a different file and located each profile’s data by linear interpolation, considering the actual profile length.

**Figure 1:**
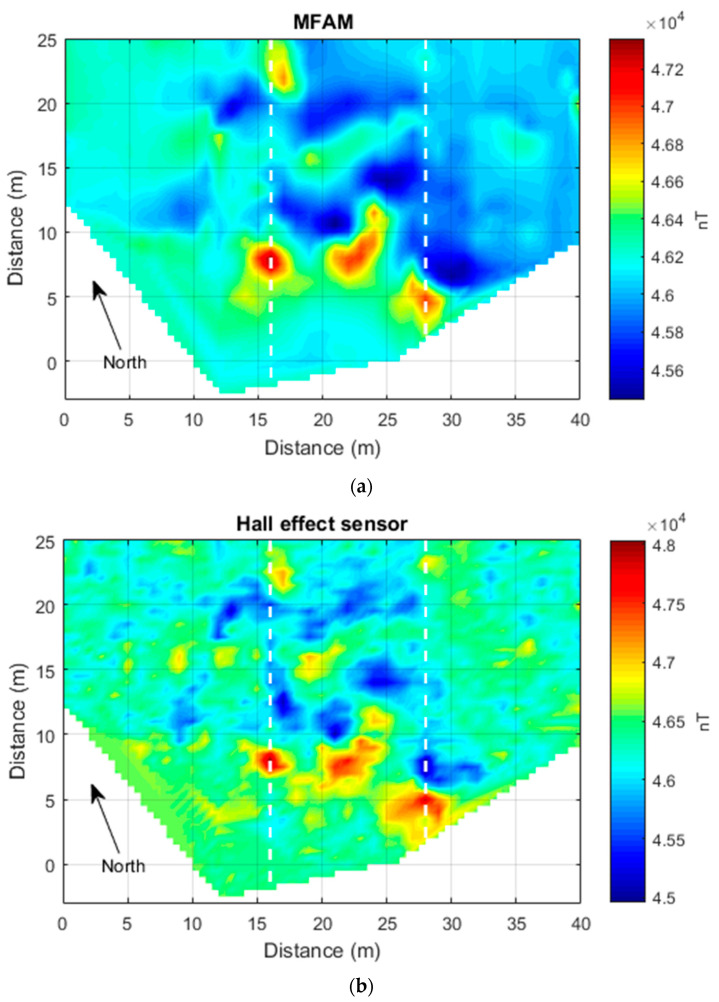
“Area 1” (Naples, Italy) case. (**a**) the Total field intensity acquired by an MFAM sensor. (**b**) The total field intensity computed by the three components of a Hall-effect sensor contained in a smartphone. Dashed lines mark the position of the profiles shown in Figure 2.

To minimize the heading error, we used a “non-meandering” acquisition mode, consisting of always starting the measurements along each profile from the north and ending in the south. The data processing consisted of a reduction in the heading error of the only Hall-effect sensor dataset, by subtracting to each profile its arithmetic mean. Then, the Hall-effect dataset average value of 46,288 nT was added back to these data. The non-meandering acquisition mode was instead sufficient to obtain a clean MFAM dataset. The two datasets were gridded using a 0.5 m sampling step (Figure 1).

The total field intensity measured by the MFAM sensor exhibits a maximum variation of about 1900 nT (Figure 1a), while the one computed from the 3-component Hall-effect sensor contained in the mobile phone (Figure 1b) has a range of about 3075 nT. This strong range difference (about 1000 nT) may be justified by the possibility of having carried the Hall-effect sensor at a slightly lower elevation with respect to the MFAM magnetometer. In fact, the magnetic field decays fast, and close to metallic objects, a difference of distance of even just 10–15 cm centimeters may produce a great change in the magnetic intensity. A simple test shows that if we model the magnetic source with a simple dipole with a magnetization sufficient to generate a total field with an amplitude of 1900 nT at a distance of 1 m, a reduction in distance of only 10 cm generates a total field with an amplitude of about 2600 nT (for a magnetization and main field directions with declination = 0° and inclination = 60°). These figures are fully comparable to our experimental data, so the amplitude difference between the two datasets can be associated with a slightly lower elevation of the Hall-effect sensor with respect to the nominal altitude of 1 m above the ground.

**Figure 2:**
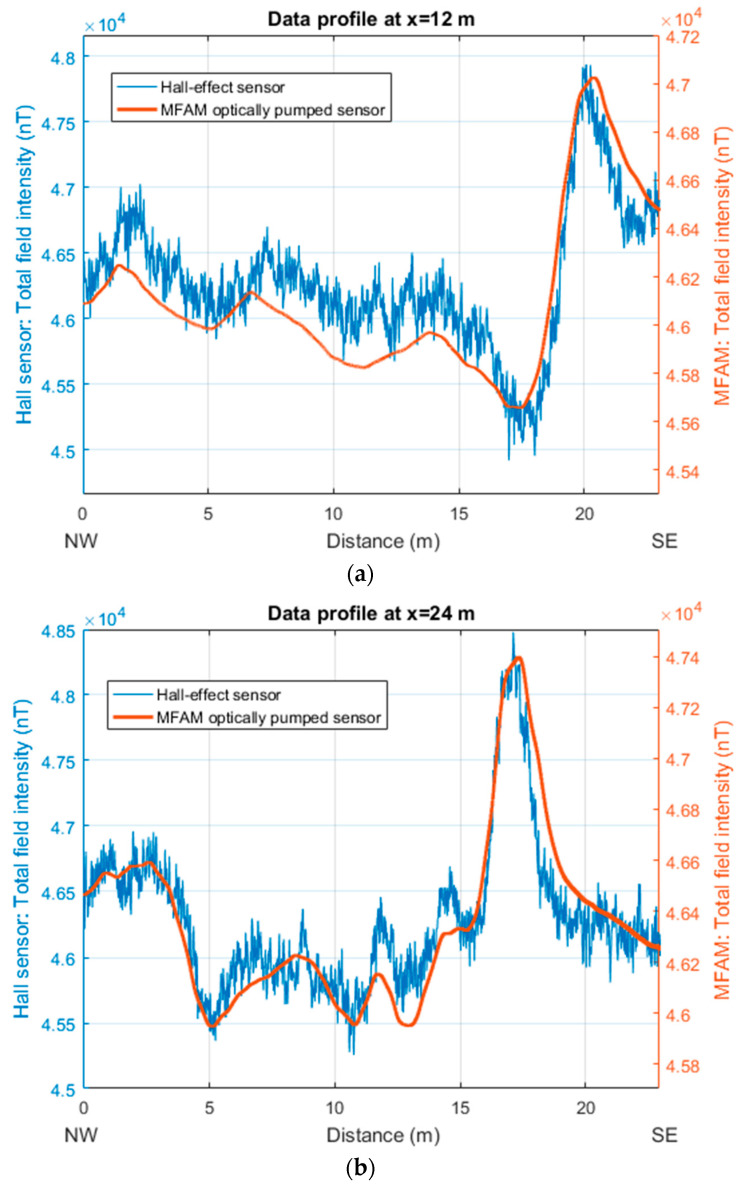
Comparison of the total field intensities recorded by an MFAM and a Hall-effect sensor at “Area 1” (Naples, Italy). (**a**) NW–SE profile located at x = 12 m in the map of Figure 1a. (**b**) NW–SE profile located at x = 24 m in the map of Figure 1b.

The two maps show the presence of intense anomalies in their central part (10 m < x < 25 m). These anomalies are mainly dipolar, including high and low areas. These highs and lows appear roughly oriented in the direction of the Earth’s magnetic field (IGRF model), which in this area has a declination of about 0° and an inclination of 56°. This may witness the presence of many highly magnetized objects, with the individual permanent magnetic moments tending to cancel out with the result of enhancing the induced magnetization effects. Overall, the MFAM data are much smoother than the Hall-effect sensor data that are affected by apparently random noise throughout the surveyed area. However, notably, the two maps give almost the same degree of information about the distribution of the magnetic anomalies in the area. This can be clearly seen when the two datasets are compared along profiles (Figure 2). This comparison shows well that the amplitude of the noise in the Hall-effect sensor data reaches 200 to 300 nT. Despite this noise, as the main anomalies in this area have a strong amplitude (>400 nT), along the two selected profiles, any anomaly visible in the MFAM data can also be identified in the Hall-effect sensor dataset (Figure 2). Other weaker anomalies visible in the MFAM map, such as those in the easternmost part of the area (x > 30 m), are instead hidden by the noise in the Hall-effect sensor map (Figure 1).

### 3.2. Area 2—Verteglia Plain

Ref. [14] presented a ground magnetic investigation in the southern Apennines (Italy), at Verteglia Plain, a small tectonic–karst depression. The magnetic survey was aimed at searching for a buried steel pipe whose existence was known, although the exact location and direction were uncertain. The survey was conducted within a 90 × 38 m^2^ area. Measurements were acquired along lines spaced 2 m apart and oriented in a north/northeast—south/southwest direction. The ground magnetic data were positioned with respect to a local reference system by introducing markers in the data stream at pre-defined positions along the profile.

In this case, we will compare the total field intensity data measured by the MFAM with those computed from the 3C AMR sensor included, with the compass function, in the control unit of the same MFAM optically pumped magnetometer. In this case, we will consider the two sets of measurements as if the sensors are co-located, although the two sensors are displaced by about 50 cm. Being different parts of the same instrument, the two sensors are carried at the same elevation above the ground.

The two raw magnetic datasets were interpolated within the survey area using a grid cell of 0.5 × 0.5 m^2^ (Figure 2). We monitored the magnetic field variations during the survey (about 2 h) and found that were negligible, so no correction was applied. The preprocessing of the two datasets was kept to a minimum, as in the previous example, and consisted only of the reduction in the heading error from the AMR sensor data by the removal of the average value along each line. Then, the average value of the magnetic field measured in the survey area by this sensor was added back (61,190 nT). The amplitude range of the magnetic anomalies of the two datasets is rather similar, about 6000 nT, although the one relative to the AMR sensor dataset is slightly larger.

**Figure 3:**
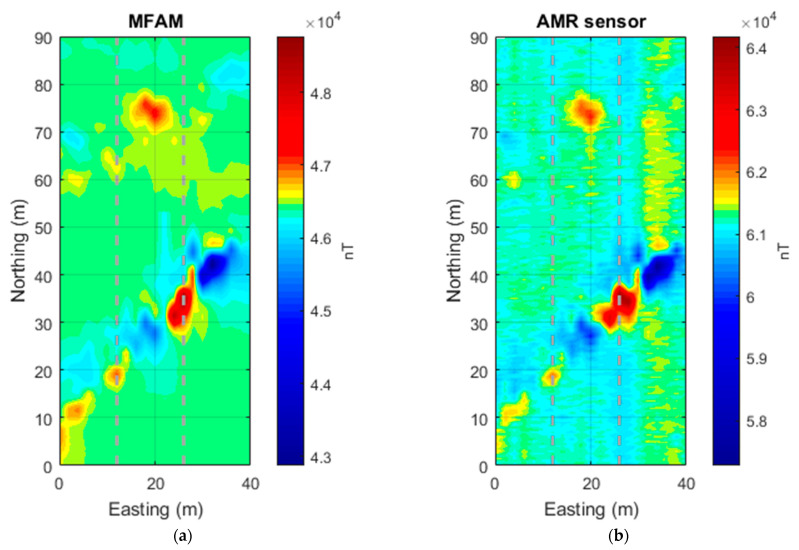
Verteglia Plain (Italy) case. (**a**) Total field intensity acquired by an MFAM sensor. (**b**) Total field intensity computed by the three components of an AMR sensor used as a compass in the MFAM. Dashed lines mark the position of the profiles shown in Figure 4. In both maps, the color bar is optimized to allow visualizing the northern low-amplitude magnetic anomalies.

A first look at the maps of Figure 3 reveals the presence of a diffuse noise in the AMR sensor dataset. The main anomalies observed in both datasets reveal the presence of a NE–SW-oriented stripe of strong magnetic anomalies in the central area (0 m < y < 50 m). These intense magnetic anomalies have the typical pattern with alternating highs and lows that can be detected above a metal pipe and that are generated by the remanent magnetization of the different sections of the pipe (e.g., [30]). Additionally, two other NE–SW anomaly alignments in the northern area (60 m < y < 85 m), approximately 10 m apart from each other, are clearly visible in the MFAM map (Figure 3a) and faintly visible in the AMR sensor map (Figure 3b). The northernmost anomaly alignment has the same “pipeline” pattern described above, but with amplitudes weaker than the anomalies in the central area. The other alignment of magnetic anomalies displays only small magnetic highs (with an amplitude of about 200 nT) and could be related to an old metallic cable (having a diameter of about 5 cm), as reported in an unpublished study from our research group. These northern anomaly alignments are clearly visible in the MFAM data, while in the AMR sensor map, it is hard to spot them, although some of the anomalies are well above the noise level.

The comparison of the two datasets along a profile allows a better assessment of the characteristics of the signals. The first profile (x = 12 m) crosses an intense anomaly at y = 20 m (with an amplitude of several hundreds of nT) belonging to the main southern alignment and a magnetic high with an approximate amplitude of about 200 nT in the north. Both datasets highlight the most intense anomaly, but the AMR data fail to show the northernmost one (at y = 65 m), which has an amplitude similar to the noise level of these data. The second profile, at x = 26 m, shows how in the two datasets, the most intense anomaly of the area is equally visible (having an amplitude of about 3000 nT).

**Figure 4:**
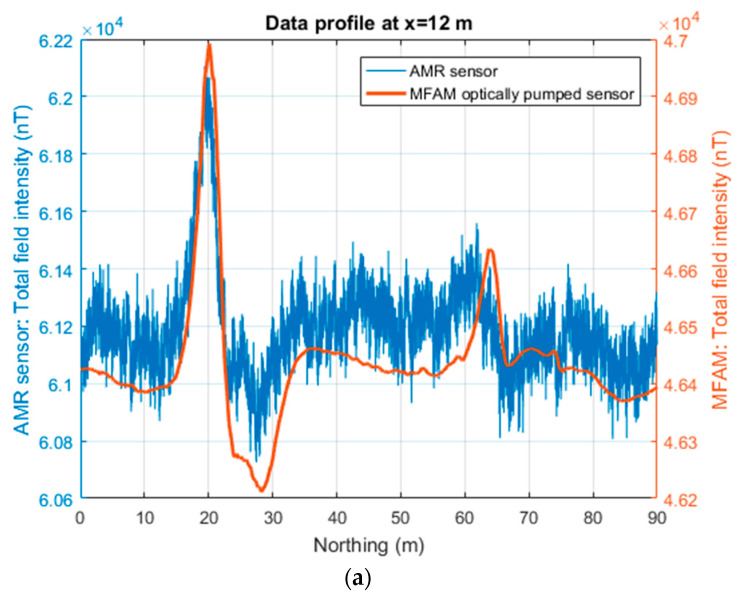

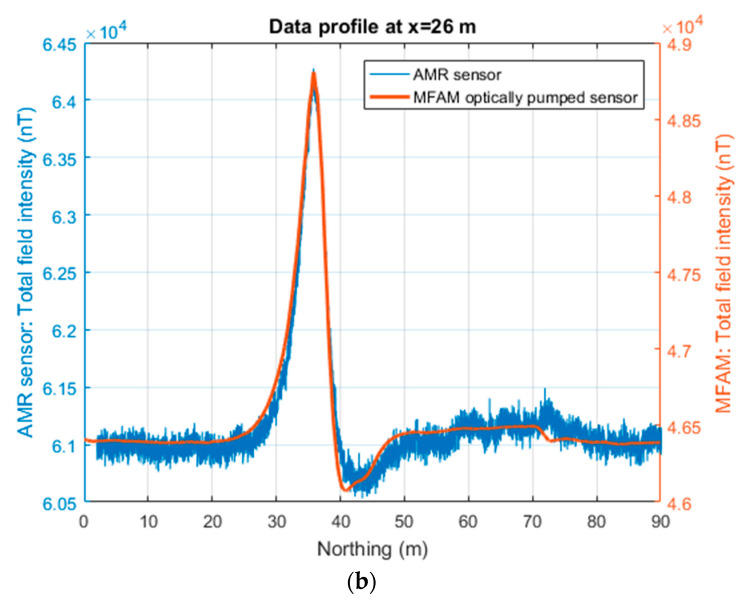
Comparison of the total field intensities recorded by an MFAM and an AMR sensor at Verteglia Plain (Italy). (**a**) S–N profile located at x = 12 m in the map of Figure 3a. (**b**) S–N profile located at x = 26 m in the map of Figure 3b.

### 3.3. Area 3—Presenzano Quarry

Like the previous case, in this last example, we compare MFAM and AMR sensor datasets acquired simultaneously as these sensors are both parts of the same instrumentation. However, with respect to the previous case, now we will describe magnetic measurements made in the context of the drone-borne survey described in [31], so the magnetic data were potentially influenced by the magnetic and electromagnetic interference due to the aerial platform and his rotors. The goal of the study was to map the areal extension of an intrusive body that emerges along the wall of an active limestone quarry.

One of the key decisions impacting data quality and the operational effectiveness of a drone-borne aeromagnetic system relates to how the magnetic sensors are fixed to the drone. To address the challenge of flying over rough and steep terrain, we decided to attach the magnetometer to the landing gear of the drone, just 0.50 m away from the motors. In this configuration, the data are affected by significant high-frequency and high-amplitude noise due to the drone and its rotors. On the other hand, this choice overcomes the problem of the noise caused by magnetometer swinging when it is connected to the UAV using long ropes. Ref. [14] demonstrated that the electric and electromagnetic noise generated by the close proximity to the drone (noise level of about 25 nT in this survey) could be well detected in the fast-sampled MFAM data (1000 Hz) and efficiently removed when strong magnetic anomalies are expected (as in the case of a shallow intrusion). However, these considerations are not tested for other sensors like the AMR sensor contained in the MFAM module, having a significantly lower bandwidth (100 Hz).

The investigated area is a limestone quarry near the town of Presenzano (southern Italy). The excavation activities have progressively exposed a mafic dike visible along the quarry wall.

The drone used for this investigation was a DJI Matrice 600 Pro hexacopter. Nine individual flights were required to cover the area (about 550 × 350 m^2^) at an elevation of 20 m AGL, and 81 parallel survey lines were flown, maintaining a horizontal separation of 10 m between them. To ensure flight safety and a consistent altitude above the terrain, flight missions were planned with a high-resolution Digital Surface Model (DSM) with a grid resolution of 1 × 1 m. The survey profiles were oriented along a north–south direction. Additionally, to mitigate heading errors, the flight paths were designed without a 180-degree turn at the end of each survey line, as recommended by [14]. We monitored the diurnal variation of the magnetic field with a GEM Overhauser magnetometer positioned near the survey area. Due to the very limited duration of the single flights (about 15 min), we recorded only negligible variations, so our processing was limited to adjust for constant offsets in the various datasets.

Despite the high-frequency noise generated by the proximity to the drone (noise frequency of about 50 Hz; [32]), to make a clearer comparison, we did not filter the datasets. Moreover, this noise has a very low amplitude with respect to the recorded magnetic anomalies and should be only barely visible after the data interpolation.

**Figure 5:**
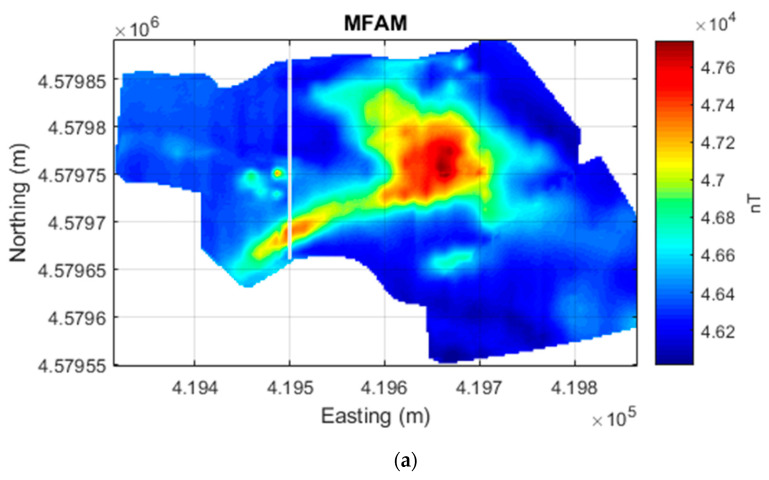

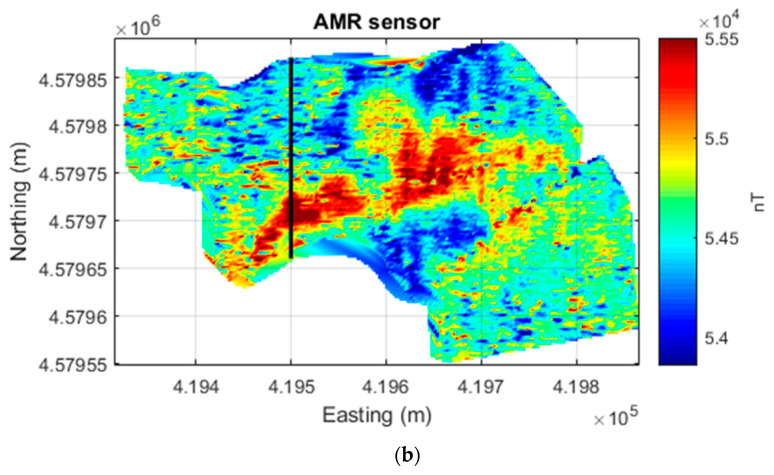
Presenzano quarry drone-borne magnetic datasets. (**a**) Total field intensity acquired by an MFAM sensor. (**b**) Total field intensity computed by the three components of an AMR sensor used as a compass in the MFAM. The line at x = 419,500 m marks the profile shown in Figure 6.

As before, the processing involved reducing the heading error from the AMR sensor data, by subtracting from each survey line its average value. Then, the average value of the entire AMR dataset was added back (54,607.7 nT). The data were interpolated on a 2.5 m × 2.5 m grid to obtain the total field intensity maps of the MFAM dataset and of the AMR dataset (Figure 5). As in the previous case of the Verteglia plain, the amplitude range of the magnetic anomalies of the two datasets is similar, about 1700 nT. The noisy character of the AMR sensor map contrasts with the smoothness of the MFAM sensor map. However, the main target of the survey, that is, the magnetic counterpart of the magmatic intrusion, is clearly evident in both maps as a SW–NE linear intense magnetic high running from the quarry area (where it outcrops) to the mountain slope. This linear magnetic anomaly assumes a rounded shape in the central part of the area, corresponding to the presence at the surface of strombolian deposits, associated with small eruptions fed by the dike [31].

**Figure 6:**
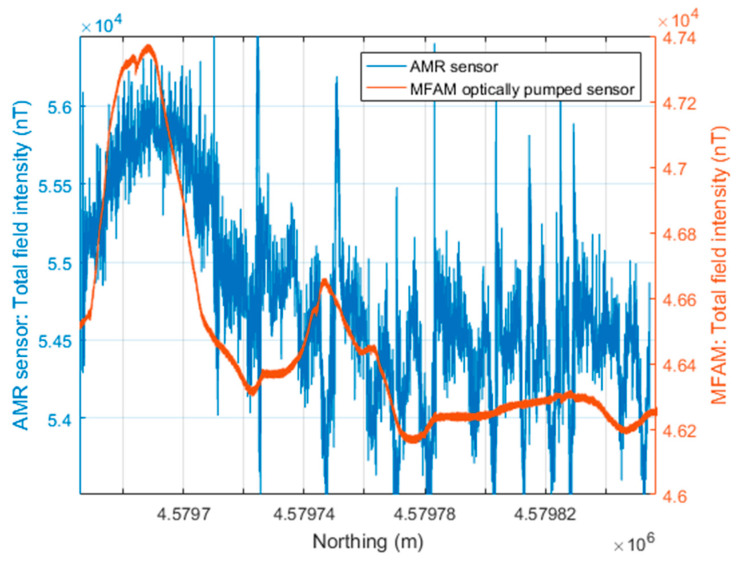
Comparison of the total field intensities recorded by an MFAM and an AMR sensor at Presenzano quarry (Italy). S–N profile located at x = 419,500 m in the map of Figure 5.

The noise level of the AMR data can be estimated at about 400 nT, as can be verified by looking at the data in more detail along a profile crossing the anomaly generated by the intrusion (Figure 6). Also evident in this dataset is the frequent presence of spikes with amplitude as strong as 1000 nT. These outliers are also very evident in the AMR sensor map (Figure 5). The MFAM profile data (Figure 6) are instead characterized by a high-frequency signal with an amplitude of about 20–25 nT generated by 50 Hz power lines and by the electric and electromagnetic noise produced by the drone, very small in the context of the target anomalies. In Figure 6, the profile crosses a magnetic anomaly caused by the presence of excavators and trucks during the survey (x = 4,579,750 m), with an amplitude of about 400 nT, which is not detected in the AMR sensor data.

## 4. Discussion

The three case histories presented above clearly show that low-cost magnetic sensors can be useful for detecting intense anomalies generated by near-surface metallic objects or even strongly magnetized geologic structures, such as a volcanic dike. This result agrees with a few previous works on this subject, described in the Introduction. Moreover, we demonstrated the possibility of recording useful magnetic data from low-cost sensors even in drone-borne surveys.

The essential limiting factor for the low-cost sensors is their low sensitivity, implying a high noise level. We verified this noise level in real data acquisition, and it varied from about 200 to 400 nT, with the most disturbed data corresponding to the drone-borne survey. We could not see strong differences between the noise levels of the two low-cost sensors tested. Differently from some previous studies [28], we did not find strong glitches or DC shifts in the low-cost sensor data presented.

Some authors (e.g., [27]) suggest setting the phone containing the Hall-effect magnetic sensor in airplane mode to avoid interference with message updates or with applications active in the background. In our case, we collected the Hall-effect sensor data shown in Figure 1 without any particular settings on the used phone.

The MFAM and Hall-effect sensors recorded an average value of the magnetic field very similar to the local IGRF intensity value (around 46,500 nT in all three surveyed areas). However, we noticed very different values for the AMR sensor datasets, namely 61,190 nT for the Verteglia plain ground data and 54,607 nT for the Presenzano drone-borne data. These higher base levels of the magnetic field might be due to disturbing static fields, as the AMR sensor we used was placed inside the MFAM control unit and relatively close to the battery pack of the instrument.

Vector datasets as recorded by the tested inexpensive sensors provide a complete three-component description of the anomalous field vector, so they carry much more information than the scalar magnetic data sensed by the optical pumping sensors. However, these data would need rather complex calibration and correction procedures to obtain a precision comparable to that of scalar magnetometers. In this paper, we did not attempt any special processing of the vector datasets other than the removal of a mean value to reduce a heading error effect. Despite this, in all the cases the results compared well with the scalar data, apart from an evidently much higher noise level. This demonstrates that if anomalous magnetic fields with intensity of hundreds of nT are considered, the vector magnetic datasets acquired with even an AMR sensor contained in a smartphone, once transformed in the total field intensity by Equation (1), can approximate well the accurate total field measured by a professional-level scalar magnetometer.

On the other hand, the amplitude of the anomalies and the signal-to-noise ratio can determine the maximum depth at which the detection of a magnetic target can be achieved. In the case of low-sensitivity sensors, this amplitude threshold is undoubtedly much higher than professional-level sensors. Thus, the limited sensing depth of low-sensitivity sensors should be shallower relative to high-sensitivity magnetometers commonly used in geophysical exploration. The above amplitude threshold may be more severe for drone-borne data, due to the higher noise level and spurious spikes so the cost of drone-mounting these low-sensitivity sensors appears to be a further degradation of performance.

The conclusion that low-cost magnetic sensors can be useful for detecting intense anomalies can be of some interest on some occasions. In the examples illustrated above, we showed their utility in selected exploration problems. Apart from many possible citizen–science applications, the widespread availability of these sensors, which are contained in every smartphone, can pave the way to innovative surveying possibilities. For example, a set of numerous smartphones carried by many operators might be used to scan very rapidly a large area in search of the effects of strongly magnetized objects. An example of practical utility might be the search for utilities such as buried cables or pipes, or the rapid search for people caught in an avalanche in an alpine environment, in case these people were equipped with a device that generates a sufficiently strong magnetic field. Another example may be the use of smartphones, even carried by small light drones, aimed at the detection of unexploded ordinance (UXO) in areas affected by war or poverty, where specialized equipment is unavailable.

## 5. Conclusions

In the past few decades, lightweight and inexpensive magnetic sensors have become available to most people—that is, to every owner of a smartphone. These sensors provide the three components of the total field, with their low sensitivity (200–400 nT) being their main limit.

The real case applications presented in this paper allowed a clear comparison of magnetic data acquired by an accurate optical pumping scalar sensor (MFAM) and inexpensive and lightweight vector magnetometers, such as Hall-effect or AMR sensors.

The datasets acquired during ground-based and drone-borne surveys over strongly magnetized sources showed that both allow the identification of the main anomalies (with amplitude of at least some hundreds of nT), so both provided useful results.

Thus, these sensors can find some useful applications within the limits of their sensitivity by helping in the exploration of highly magnetized volcanic structures or in the search for metallic objects. On the other hand, the high noise level characteristic of Hall-effect or AMR sensors could prevent accurate quantitative modeling of the anomalies, so such data should be considered only in selected applications and intended as a reconnaissance tool. However, the widespread diffusion of such sensors may open the space for possible innovative ways to perform a magnetic survey in some exploration cases.

## Data Availability

The datasets presented in this article are not readily available because the data are part of an ongoing study.
